# THE ASSOCIATION BETWEEN MODERATE-TO-VIGOROUS PHYSICAL ACTIVITY AND MUSCLE OXIDATIVE CAPACITY IN OLDER ADULTS

**DOI:** 10.1093/geroni/igz038.326

**Published:** 2019-11-08

**Authors:** Fatemeh Adelnia, Jacek Urbanek, Yusuke Osawa, Michelle Shardell, Eleanor M Simonsick, Jennifer A Schrack, Luigi Ferrucci

**Affiliations:** 1 National Institute of Health, Baltimore, Maryland, United States; 2 John Hopkins University School of Medicine, Department of Medicine, Division of Geriatric Medicine and Gerontology, Center on Aging and Health, Baltimore, Maryland, United States; 3 Translational Gerontology Branch, Intramural Research Program, National Institute on Aging, National Institutes of Health, Baltimore, Maryland, United States; 4 Longitudinal Studies Section, Intramural Research Program, National Institute on Aging, Baltimore, Maryland, United States; 5 Johns Hopkins University, Baltimore, Maryland, United States

## Abstract

Age-related decline in muscle oxidative capacity negatively affects muscle function and mobility, which may lead to disability and frailty. Whether exercise and other life-style practices reduce age-related decline in muscle oxidative capacity is unclear. We assessed whether, after accounting for age, higher daily physical activity levels are associated with greater muscle oxidative capacity. Participants included 384 adults (54.7% women) aged 22 to 92 years from the Baltimore Longitudinal Study of Aging. Muscle oxidative capacity was measured in vivo using phosphorous magnetic resonance spectroscopy. We determined the time constant for phosphocreatine recovery (τPCr, in seconds) after exercise, with lower values of τPCr reflecting greater oxidative capacity. Time spent in moderate-to-vigorous physical activity (MVPA) was assessed using accelerometers that participants wore for 5.9 ± 0.9 consecutive days in the free-living environment. In linear regression models, older age was associated with higher τPCr (β = 0.39, p-value <.001) after adjusting for sex, race, height and weight. After including MVPA as an independent variable, the standardized regression coefficient for age was attenuated by 40% to 0.22. p-value <.001). MVPA was strongly associated with lower τPCr (β = -0.33, p-value <.001) after adjusting for health status, education and smoking history and was only attenuated by 3% after additional adjustment for age. These results suggest that MVPA is strongly associated with muscle oxidative capacity independent of age, providing mechanistic insights into the health benefits of daily physical activity in older persons.

